# Controlling the length scale and distribution of the ductile phase in metallic glass composites through friction stir processing

**DOI:** 10.1088/1468-6996/15/3/035011

**Published:** 2014-06-24

**Authors:** Harpreet Singh Arora, Sanghita Mridha, Harpreet Singh Grewal, Harpreet Singh, Douglas C Hofmann, Sundeep Mukherjee

**Affiliations:** 1 Department of Materials Science and Engineering, University of North Texas, Denton, Texas 76203, USA; 2 School of Mechanical, Materials and Energy Engineering, Indian Institute of Technology Ropar, Rupnagar, Punjab 140001, India; 3 Engineering and Science Directorate, Jet Propulsion Laboratory, California Institute of Technology, Pasadena, CA 91109, USA

**Keywords:** bulk amorphous alloys, thermomechanical processing, nanoindentation, shear bands

## Abstract

We demonstrate the refinement and uniform distribution of the crystalline dendritic phase by friction stir processing (FSP) of titanium based *in situ* ductile-phase reinforced metallic glass composite. The average size of the dendrites was reduced by almost a factor of five (from 24 *μ*m to 5 *μ*m) for the highest tool rotational speed of 900 rpm. The large inter-connected dendrites become more fragmented with increased circularity after processing. The changes in thermal characteristics were measured by differential scanning calorimetry. The reduction in crystallization enthalpy after processing suggests partial devitrification due to the high strain plastic deformation. FSP resulted in increased hardness and modulus for both the amorphous matrix and the crystalline phase. This is explained by interaction of shear bands in amorphous matrix with the strain-hardened dendritic phase. Our approach offers a new strategy for microstructural design in metallic glass composites.

## Introduction

Metallic glasses are an exciting class of materials with very high strength and perfectly elastic behavior. However, limited plasticity and catastrophic failure have restricted their widespread use in structural applications. Therefore, the design and development of new metallic glass alloys are driven by improvement in ductility and fracture toughness for load-bearing applications [1, 2]. To counter limited plasticity, ductile-phase reinforced metallic glass composites have been developed [1–6]. Introducing inhomogeneity at the microstructural length scale stabilizes against unlimited extension of shear bands and catastrophic failure [6, 7]. Recently, the development of a titanium-based metallic glass composite with exceptionally high tensile ductility (>10%) and improved fracture toughness has been reported [1]. The improvement in tensile ductility for metallic glass composites results from the arrest of shear bands by the elastic dendritic phase. The shear band pattern surrounding the dendrite domains in metallic glass composites prevents the formation of opening cracks, which results in high toughness and global plasticity [1]. Therefore, size and distribution of the ductile crystalline phase significantly influence the properties of these composites. Microstructural refinement comprising fragmentation and homogeneous distribution of the ductile phase can further improve the properties of metallic glass composites. Severe plastic deformation (SPD) processes are widely used for microstructural refinement in crystalline materials. Friction stir processing (FSP) is one such SPD process [8, 9].

In this paper, we report on the structural changes in an *in situ* ductile-phase reinforced metallic glass composite (Ti_48_Zr_20_V_12_Cu_5_Be_15_) after friction stir processing. This composite has one of the highest specific strength of all known materials (>300 MPa cm^−3^ g^−1^) as well as high room-temperature tensile ductility [1]. FSP was done with two different conditions of 500 rpm and 900 rpm tool rotational speeds. FSP has several important consequences—it leads to refinement and more uniform distribution of the ductile dendrite phase. In addition, there is change in hardness and modulus of the amorphous matrix as well as the *in situ* crystalline phase. We discuss a mechanism to account for the observed change in mechanical and thermophysical behavior based on shear band interaction with the crystalline phase. Our approach offers a novel strategy in microstructural design using FSP for a wide range of metallic glass compositions. This is potentially transformative in the control of length scale and distribution of crystalline phase in a metallic glass composite.

## Experimental procedures

**Figure 1:**
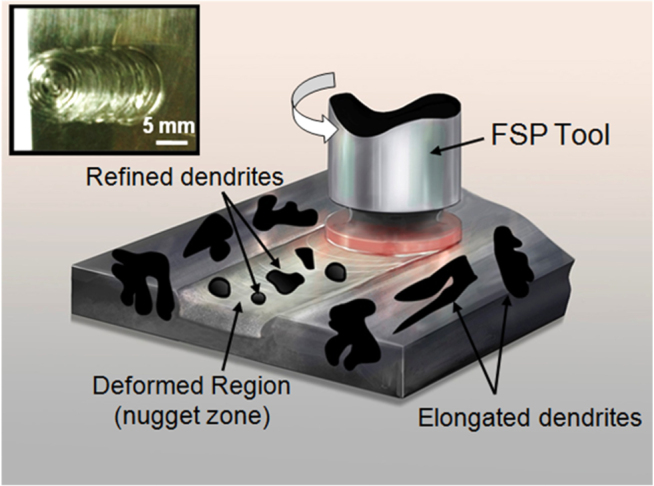
Schematic of friction stir processing (FSP). The underlying material is subjected to high strain during FSP resulting in fragmentation and homogeneous distribution of the dendrites. The inset shows the actual image of the nugget zone of friction stir processed metallic glass. The highly strained material in the nugget zone comprises fine dendrites compared to elongated ones in the un-deformed region. All samples for thermal analysis and nano-indentation were taken from the center of the nugget zone to eliminate any non-uniformity and edge effects.

The material used in the current study is a titanium-based metallic glass composite, Ti_48_Zr_20_V_12_Cu_5_Be_15_. This composite has nearly 47% volume fraction of ductile dendritic phase distributed in the amorphous matrix. FSP was performed on a computer numerical control vertical milling machine. The FSP tool used was pin-less with shoulder diameter of 10 mm. The FSP parameters comprise two different tool rotational speeds of 500 rpm and 900 rpm, and a plunge depth of 0.3 mm. A schematic for FSP of the composite is shown in figure 1. The elongated and inter-connected dendrites in the starting material get refined and the dendrite circularity (*C*) increases after FSP, where 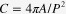, *A* is the area, and *P* is the perimeter. An image of the nugget zone for the processed metallic glass composite is shown in the inset of figure 1. Temperature of the surface during FSP was measured using a K-type thermocouple. Microstructural studies were performed using scanning electron microscopy (SEM). To measure the grain size of the crystalline dendritic phase, samples were etched using Kroll’s reagent and the microstructure was observed using SEM. Differential scanning calorimetry (DSC) was used to determine glass transition temperature (*T*_*g*_), crystallization temperature (*T*_*x*_), and enthalpy changes. The hardness and modulus were obtained using nano-indentation on the top surface of all the specimens. The reported values for hardness and modulus are the average of ten readings for each specimen. Testing was done at a peak load of 10 mN using a standard Berkovich tip. All the samples obtained for the DSC and nano-indentation test were taken from the center of the nugget zone to eliminate any non-uniformity and edge effects. High-resolution transmission electron microscopy (HRTEM) was used to analyze the structure of as-cast and the processed samples.

## Results and discussion

**Figure 2:**
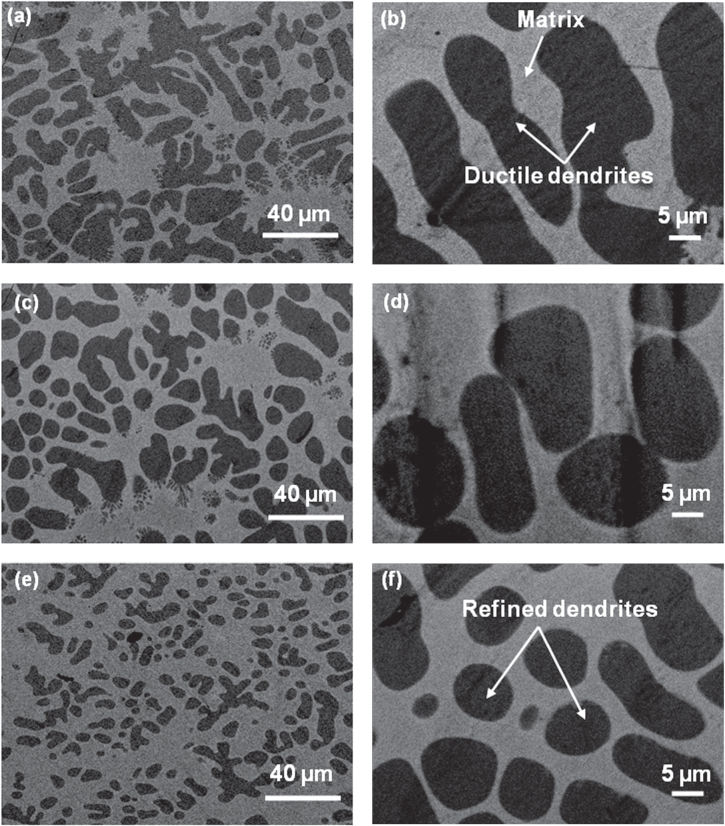
(a) Low magnification and (b) high magnification back-scattered SEM images of as-cast metallic glass composite, Ti_48_Zr_20_V_12_Cu_5_Be_15_; (c) low magnification and (d) high magnification back-scattered SEM images of metallic glass composite friction stir processed at 500 rpm (FSP 500); (e) low magnification and (f) high magnification back-scattered SEM images of metallic glass composite friction stir processed at 900 rpm (FSP 900). The composite microstructure comprise ductile dendritic phase distributed in the amorphous matrix. The dendrites get fragmented during friction stir processing and are more uniformly distributed in the amorphous matrix.

**Figure 3:**
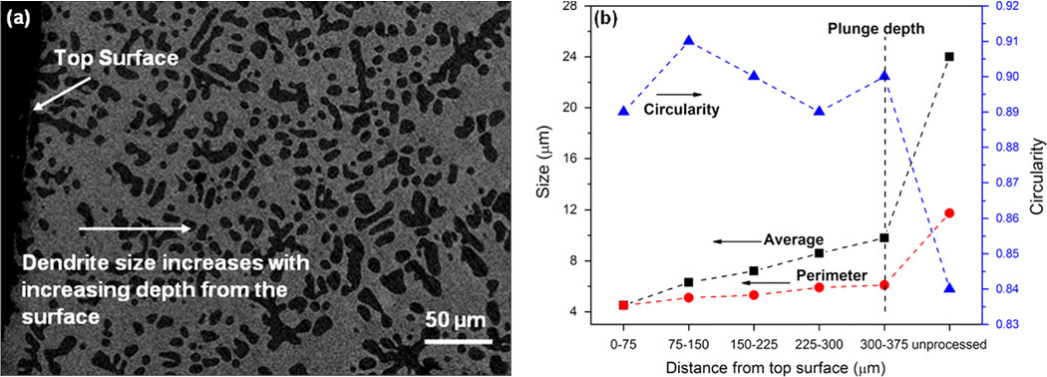
(a) SEM cross-section image of metallic glass composite friction stir processed at 900 rpm (FSP 900); (b) dendrite size distribution and particle circularity along the cross-section as a function of distance from the top surface. The dendrites are finer near the top surface and increase almost linearly with depth upto the plunge depth of the FSP tool. The circularity of particles also increases after friction stir processing due to their fragmentation. The solid symbols represent the data points, while the connecting lines help in reading the trend.

Back-scattered SEM images of the as-cast metallic glass composite, composite friction stir processed at 500 rpm (FSP 500) and 900 rpm (FSP 900) are shown in figures 2(a)–(f). The as-cast microstructure consists of 47 vol % of body centered cubic dendritic phase with composition Ti_66_V_19_Zr_14_Cu_1_ (excluding Be, which cannot be measured by energy-dispersive x-ray spectroscopy but is known to be < 3 at.%), and 53 vol % of amorphous matrix with approximate composition Ti_32_Zr_25_V_5_Cu_10_Be_28_ [1]. Using ImageJ software, the average size of the dendritic phase was found to be nearly 24 *μ*m. The dendrites get fragmented during FSP as shown in figures 2(c)–(f). The back-scattered SEM image of the FSP 900 specimen cross-section is shown in figure 3(a). The size distribution of the dendrites at increasing depth from the surface along the specimen cross-section is given in figure 3(b). The figure shows an average size modeled using an ellipse fit, the perimeter as well as the dendrite circularity. It is seen that the dendrites are finer near the top surface of the specimen with an average size of nearly 4.5 *μ*m. The dendrite size increases almost linearly with increasing depth upto 375 *μ*m, which corresponds to the plunge depth of the FSP tool. The peak temperature for FSP 500 and FSP 900 specimens was measured to be nearly 300 °C and 450 °C, respectively. The solidus temperature of the composite (∼682 °C) [1] is significantly higher compared to the peak temperatures reached during processing. Therefore, the mechanism for dendrite refinement is likely to be mechanical fragmentation from the high-strain deformation process rather than dendrite dissolution. The degree of strain increases with the increase in FSP tool rotational speed, which explains the greater refinement of the dendrites at 900 rpm. The strain rate during FSP is higher near the tool shoulder owing to the material’s higher rotational velocity [10], which explains the smaller dendrites near the top surface. Further, the particle circularity also increases during FSP due to their fragmentation (figure 3(b)).

**Figure 4:**
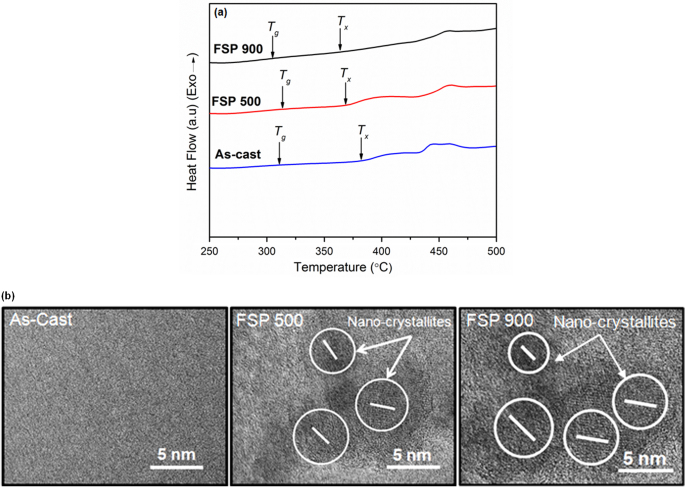
(a) DSC curves for the as-cast metallic glass composite, metallic glass composite friction stir processed at 500 rpm (FSP 500), and at 900 rpm (FSP 900); (b) HRTEM images for matrix of the as-cast metallic glass composite, FSP 500 and FSP 900 specimens. As-cast metallic glass composite as well as the processed specimens show two crystallization peaks. Glass transition temperature and crystallization temperature are indicated by *T*_*g*_ and *T*_*x*_ respectively. Matrix of as-cast metallic glass composite has a fully amorphous structure, whereas nano-crystallites can be seen in processed specimens indicating partial devitrification. The lines indicate the directionality.

To determine the changes in the thermal characteristics of the amorphous matrix after processing, DSC measurements were carried out. DSC curves for all specimens are shown in figure 4(a). All samples for thermal analysis were taken from the center of the nugget zone to eliminate any non-uniformity and edge effects. The glass transition temperature (*T*_*g*_), crystallization temperature (*T*_*x*_), and enthalpy of crystallization are given in table 1. All DSC curves show two crystallization peaks. The crystallization enthalpies for both the processed specimens are lower compared to the as-cast metallic glass composite. HRTEM images of the matrix of as-cast metallic glass composite, FSP 500 and FSP 900 specimens are shown in figure 4(b). The as-cast specimen has a fully amorphous structure, whereas nano-crystallites are clearly seen in the microstructure of both the processed specimens, indicating partial devitrification of the matrix. The crystallite size is on the order of a few nanometers. The microstructure for both the processed specimens is similar with FSP 900 having higher volume fraction of the nano-crystallites. Thus, the lower crystallization enthalpy for the processed specimens may be due to the partial devitrification during FSP. The crystallization enthalpy for FSP 900 specimen is roughly one-third of the as-cast composite indicating that a large fraction of the amorphous matrix crystallized at 900 rpm. This may be attributed to the high strain plastic deformation during FSP, which is known to induce crystallization in metallic glasses [11].

**Table 1.** DSC analysis comprising glass transition temperature (*T*_*g*_), crystallization temperature (*T*_*x*_), and crystallization enthalpy (*ΔH*) for as-cast metallic glass composite (as-cast), metallic glass composite friction stir processed at 500 rpm (FSP 500), and 900 rpm (FSP 900).

Specimen	*T*_*g*_ (°C)	*T*_*x*_ (°C)	*ΔH* (J gm^−1^)
As-cast	311	382	34.47
FSP 500	314	369	30.87
FSP 900	307	365	13.32

**Figure 5:**
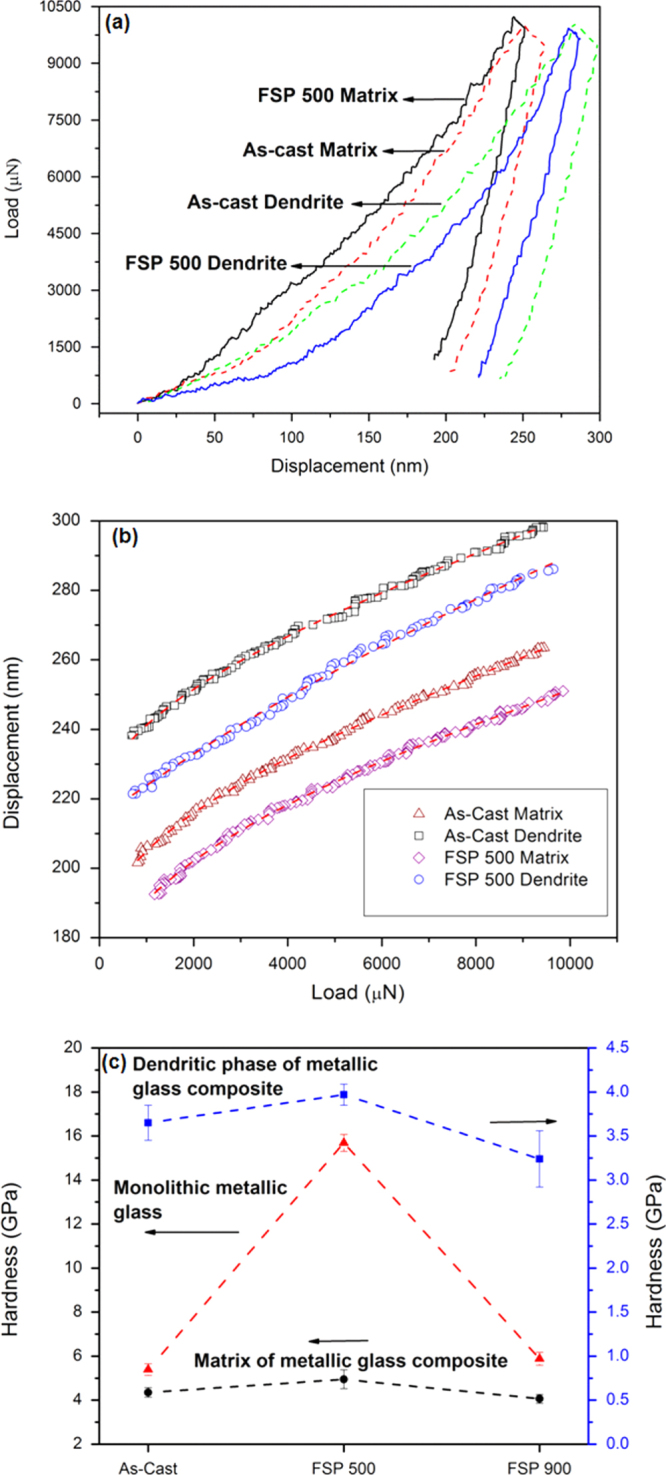
(a) Load-displacement curves for the matrix and dendritic phase in the as-cast metallic glass composite and FSP 500 specimens; (b) curve fitting for the unloading part of the load-displacement curve for the matrix as well as dendritic phase of the as-cast and FSP 500 specimens; (c) hardness variation for the matrix and ductile phase of as-cast metallic glass composite (as-cast), FSP 500, and FSP 900 specimens. Values for a monolithic metallic glass are also shown. Matrix shows smaller indentation depth indicating higher hardness than the dendritic phase. Matrix as well as dendritic phase in FSP 500 specimen shows higher hardness compared to as-cast metallic glass composite. Monolithic metallic glass as well as metallic glass composite show similar hardness variation at different FSP tool rotational speeds.

We investigated the change in hardness and modulus of each phase separately by nano-indentation. The load-displacement curves for the matrix and ductile dendrite phase for the as-cast metallic glass composite and FSP 500 specimen are shown in figure 5(a). The FSP 900 specimen shows very similar load-displacement curves, which have not been included for better clarity of the figure. The serrations seen in the load-displacement curves are typical for metallic glasses and characterize localized plastic deformation processes in the form of shear band formation and propagation [12, 13]. The indentation depth is smaller for the matrix phase compared to dendrites indicating its higher hardness and modulus. The load-displacement curves for all specimens show creep during the hold at maximum load. Creep displacement affect the modulus values and can be determined from the creep factor, 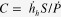, where 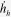 is the indenter displacement rate during hold at maximum load, *S* is the contact stiffness and 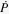 is the unloading rate at the onset of unload [14]. In the current study, the creep factor was found to be greater than 10% (*C* > 10%) and therefore likely to influence the modulus value [14]. For significant creep displacement, the usual power law given by Oliver and Pharr cannot provide a good fit for the unloading curve. The equation, 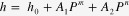, where 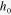, 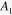, 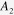, *m* and *n* are the fitting constants, gives a better fit for the onset of the unloading curve [14]. The unloading curves for all the specimens were fitted using the above equation and is shown in figure 5(b). The contact stiffness was determined for the matrix and the dendritic phase of the as-cast as well as processed specimens from the fitted unloading curves and creep-corrected modulus values were calculated. The results for nano-indentation and creep-corrected modulus values are summarized in table 2. It is seen that for the FSP 500 specimen, both the matrix and dendrite show higher hardness and modulus compared to as-cast metallic glass composite. Figure 5(c) shows the variation in hardness values for the matrix and dendritic phase of as-cast and friction stir processed composites. A monolithic metallic glass, Zr_44_Ti_11_Cu_10_Ni_10_Be_25_, was also friction stir processed under similar conditions. The hardness variation for the as-cast as well as processed monolithic metallic glass is given in figure 5(c), which shows a similar trend as the metallic glass composite.

**Table 2.** Nano-indentation results comprising hardness (*H*) and modulus values (*E*) for the matrix and dendrite phase in as-cast metallic glass composite (as-cast), metallic glass composite friction stir processed at 500 rpm (FSP 500), and 900 rpm (FSP 900). The creep-corrected modulus values (*E*_corrected_) are also given.

	As-cast		FSP 500		FSP 900	
Specimen	Matrix	Dendrite	Matrix	Dendrite	Matrix	Dendrite
*H* (GPa)	4.4 ± 0.2	3.7 ± 0.2	5.0 ± 0.4	4.0 ± 0.1	4.1 ± 0.2	3.2 ± 0.3
*E* (GPa)	114 ± 10	105 ± 9	117 ± 8	110 ± 10	107 ± 10	96 ± 3
*E*_corrected_ (GPa)	97 ± 5	83 ± 4	106 ± 5	91 ± 5	85 ± 4	78 ± 4

**Figure 6:**
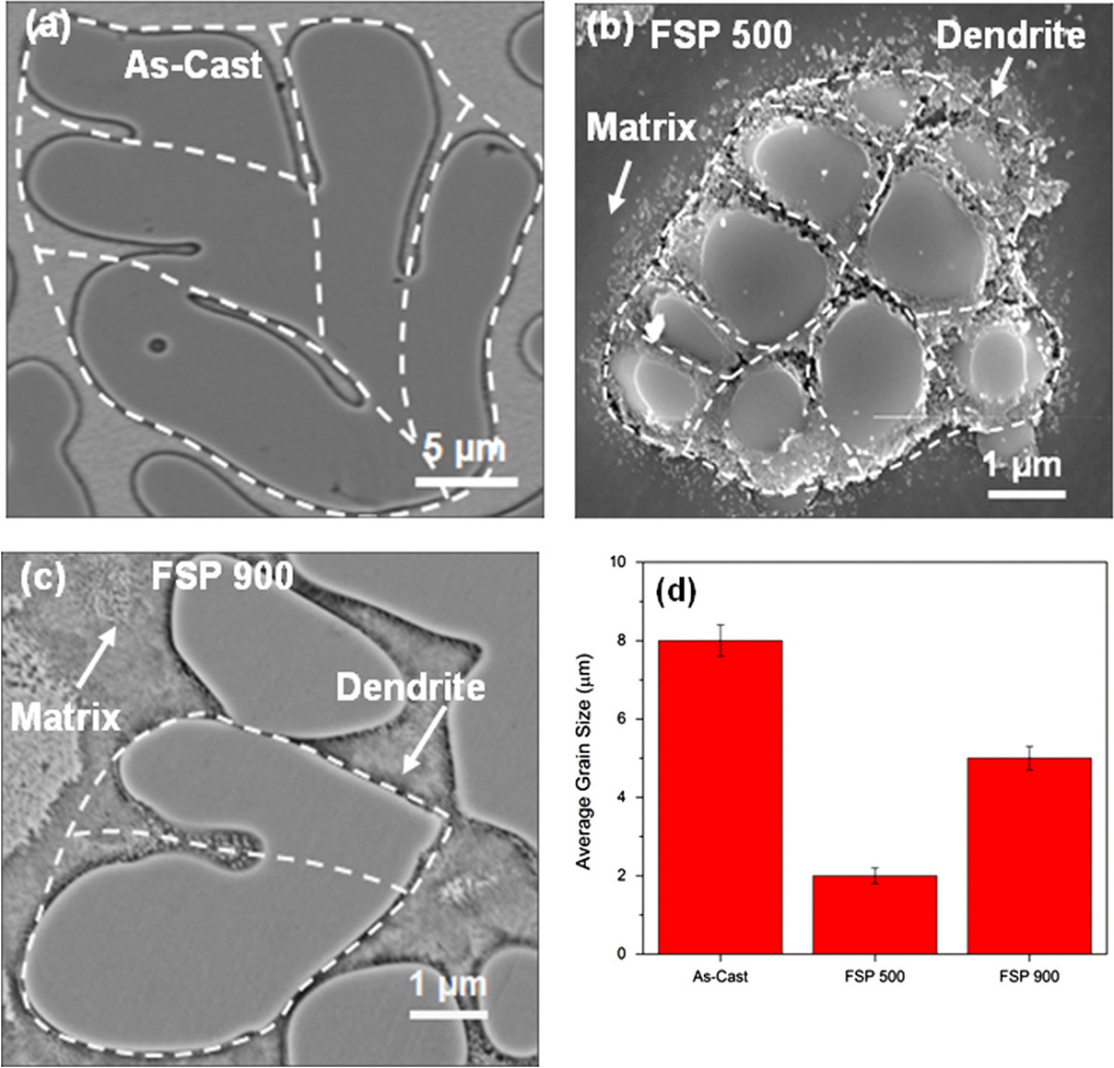
Grain structure and the average grain size for the crystalline dendritic phase of the as-cast composite, FSP 500 and FSP 900 specimens. Grain boundaries, revealed after etching with Kroll’s reagent, are indicated by dotted lines as a guide to the eye.

FSP resulted in partial devitrification of the amorphous matrix as seen from DSC curves and TEM images, along with fragmentation of the crystalline dendritic phase in the composite (figures 2 and 3). The crystalline phase contributes towards strengthening in metallic glasses by interaction among shear bands and crystallites [1, 11, 15]. In the current composite, the shear modulus for the dendrite phase is significantly lower than the matrix [1]. Therefore, shear bands likely propagate towards the softer crystalline phase [16]. In a tensile test, this metallic glass composite showed a large number of serrations in the stress–strain curve just before failure. The composite showed a total elongation of nearly 12.5% with significant work hardening [1]. The presence of serrations in the tensile test curve and significant work hardening shown by this composite indicates extensive shear band stabilization. Therefore, the homogeneously distributed dendrites in processed composite act as barriers to the deformation induced shear bands through shear band and dendrite interaction. Further, the intersecting shear band pattern around the dendritic domain [1] contributes to hardening through shear band and shear band interaction. Therefore, higher hardness and modulus of the matrix in the FSP 500 specimen is likely from these restrictive interactions. With further increase in crystalline volume fraction at 900 rpm, the properties of the amorphous matrix deteriorated. Figure 6 shows the grain structure and the average grain size for the crystalline dendritic phase of the as-cast and processed specimens. The average grain size was found to be about 8 *μ*m for the as-cast composite, and it reduces to 2 *μ*m for FSP 500 specimen. For a higher tool rotational speed of 900 rpm, the grain size increased to 5 *μ*m. For crystalline materials, the size of the recrystallized nuclei during plastic deformation decreases with the increase in strain, following the relation, 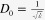, where 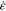 is the strain rate [17]. High strain rates during FSP promote recrystallization and nucleation of fine grains in crystalline materials. Higher hardness of the crystalline phase for the FSP 500 specimen may be attributed to its refined grain size. Increase in hardness with grain size refinement during FSP has been shown in earlier studies as well [18, 19]. However, further increase in tool rotational speed resulted in grain coarsening due to larger heat input and higher temperature. Lower hardness and modulus for the crystalline phase in the FSP 900 specimen is likely from the coarser grain structure. Decrease in hardness at higher tool rotational speed has been reported for a number of crystalline materials subjected to FSP [20–22].

## Conclusions

We demonstrated a mechanism to control the length scale and distribution of ductile phase in a metallic glass composite. FSP can be effectively used for localized microstructural transformation and applied to a wide range of metallic glass compositions. Fragmentation of the ductile crystalline phase during FSP results in a very homogeneous distribution in the composite. The control over length scale, distribution, and mechanical behavior can be enhanced by using tool rotational speed as a processing parameter to realize the requirement for a wide range of applications.
